# Vascular Instability and Neurological Morbidity in Sickle Cell Disease: An Integrative Framework

**DOI:** 10.3389/fneur.2019.00871

**Published:** 2019-08-13

**Authors:** Hanne Stotesbury, Jamie M. Kawadler, Patrick W. Hales, Dawn E. Saunders, Christopher A. Clark, Fenella J. Kirkham

**Affiliations:** ^1^Developmental Neurosciences, UCL Great Ormond Institute of Child Health, London, United Kingdom; ^2^Department of Radiology, Great Ormond Hospital, London, United Kingdom; ^3^Clinical and Experimental Sciences, University of Southampton, Southampton, United Kingdom; ^4^Department of Child Health, University Hospital Southampton, Southampton, United Kingdom; ^5^Department of Paediatric Neurology, Kings College Hospital NHS Foundation Trust, London, United Kingdom

**Keywords:** sickle cell disease, stroke, silent cerebral infarction, cerebral hemodynamics, vascular instability, anemia, oxygen extraction fraction, cerebrovascular reserve

## Abstract

It is well-established that patients with sickle cell disease (SCD) are at substantial risk of neurological complications, including overt and silent stroke, microstructural injury, and cognitive difficulties. Yet the underlying mechanisms remain poorly understood, partly because findings have largely been considered in isolation. Here, we review mechanistic pathways for which there is accumulating evidence and propose an integrative systems-biology framework for understanding neurological risk. Drawing upon work from other vascular beds in SCD, as well as the wider stroke literature, we propose that macro-circulatory hyper-perfusion, regions of relative micro-circulatory hypo-perfusion, and an exhaustion of cerebral reserve mechanisms, together lead to a state of cerebral vascular instability. We suggest that in this state, tissue oxygen supply is fragile and easily perturbed by changes in clinical condition, with the potential for stroke and/or microstructural injury if metabolic demand exceeds tissue oxygenation. This framework brings together recent developments in the field, highlights outstanding questions, and offers a first step toward a linking pathophysiological explanation of neurological risk that may help inform future screening and treatment strategies.

## Introduction

Sickle cell disease (SCD) refers to a group of inherited hemoglobinopathies that affect ~20–25 million people globally (1, 2). The condition is caused by a single-base substitution that leads to the production of mutant hemoglobin type S (HbS). When oxygen tension is low, HbS polymerizes, giving erythrocytes their characteristic “sickle” shape. The wider pathophysiology is complex and appears to involve a cycle of inter-related processes, including erythrocyte-leukocyte adhesion to the endothelium, endothelial activation, hemolysis, inflammation, and hyper-coagulation (3–8).

## Neurological Complications

**Figure 1:**
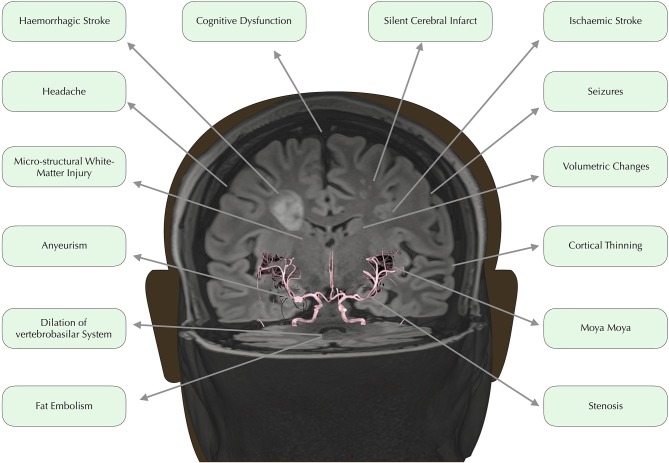
Neurological complications. Time of flight angiography image overlaid on 3D rendered Fluid Attenuated Inversion Recovery (FLAIR) image, edited to depict common neurological complications in SCD.

In developed countries, medical advances have led to dramatically increased life expectancy for children with SCD (9). The transition from fatal disorder to chronic illness has, however, brought a new set of challenges with regard to the clinical complications that can have major implications for quality of life. Among the most debilitating and poorly understood complications are a number of conditions affecting the brain, including overt and silent stroke, cerebrovascular disease, cognitive impairment, and structural abnormalities (Figure 1). Below, we consider these in turn.

## Stroke and Cerebrovascular Disease

In the absence of screening and prophylactic treatment (10), ~11% of SCD patients will suffer an overt stroke by their 20th birthday, and 24% by their 45th (11). Ischemic insults are most common, accounting for up to 75% of SCD-related strokes (12, 13). Patients are however at considerable risk of both overt ischemic and hemorrhagic stroke, with the former reported more frequently in children, and the latter more frequently in young adults (14–16). In a recent cohort study, 10% of SCD patient deaths were attributable to overt stroke (17). Whilst overt ischemic stroke is rarely fatal, death may occur following 26% of hemorrhagic cases (11). Without secondary prevention, recurrence rates of up to 70% have been reported for overt ischemic stroke, with the risk greatest within 36 months of the initial event (18). Both types of overt stroke are associated with significant long-term morbidity, including seizures, physical disability, and cognitive impairment (19).

More common than overt stroke is “silent cerebral infarction” [SCI; (20)], where hyperintensities consistent with infarction/ischemia are apparent on brain MRI in the absence of focal neurological symptoms. SCI may occur as early as the 6th month of life (21, 22). There is evidence that prevalence reaches 25% by 6 years of age (23), 39% by 18 years of age (24), and 53% by young adulthood (25), with no reports of a plateau. Although clinically “silent,” evidence of progression was first provided by the co-operative study of SCD (CSSD), where SCI was associated with a 14-fold increase in risk of overt ischemic stroke, and 25% of children with SCI presented with new or enlarged lesions at follow-up (26). In the CSSCD, SCI was also associated with cognitive decline (27). These findings have been replicated in more recent work, including in a study where SCI in patients younger than 5 years old were shown to be associated with later progressive ischemia, vasculopathy, academic difficulties, and a higher risk of overt ischemic stroke (21). Further indicative of progressive ischemia, a recent clinical review of 60 unselected adult cases found that 37% of patients with SCI had more than one lesion (25).

Infarction in the territory of large intracranial vessels is the most common pattern in SCD patients with overt ischemic stroke, but the watershed regions of the deep white-matter are particularly vulnerable (16, 28, 29), whether or not there is concomitant intra-cranial cerebral vasculopathy (30). The distribution of SCI is similar, with up to 90% of SCI reportedly occurring in a relatively small deep watershed white matter region, encompassing only 5.6% of brain volume (31). SCI and overt ischemic stroke are often indistinguishable on MRI (32), and several authors have suggested that it may be differences in lesion size and location, rather than underlying physiological mechanism, that determines whether an ischemic insult is accompanied by focal symptoms (ischemic stroke) or goes undetected [SCI; (33)].

Both in addition to, and in the absence of, overt stroke and SCI, vasculopathy on MR angiography (MRA) is common in SCD patients (32). Although vasculopathy definitions have varied considerably between studies, intra- and extra- cranial steno-occlusive arteriopathy, often involving the distal internal carotid and the proximal anterior and middle cerebral arteries, are frequently reported, particularly in patients with overt ischemic stroke (34, 35), and SCI (24). Incidence of progressive stenosis with compensatory collateral vessel formation is as high as 30–40% in SCD patients with vasculopathy (36, 37). In a multi-center pediatric study in which 37 chronically transfused patients underwent serial MRI, 38% of patients presented with a new vessel segment of stenosis or occlusion at follow-up (38). Despite aggressive hematological management, the children with vasculopathy progression were also 12 times more likely to present with new SCI or overt ischemic stroke than those with no progression.

Some authors have proposed a sequential moyamoya-like model of SCD vasculopathy and stroke (39), in which early ischemic events are associated with stenosis, and later hemorrhagic events with the development and eventual rupturing of friable and maximally dilated collateral vessels. However, the majority of SCD-related intra-cerebral and subarachnoid hemorrhages are associated not with collateral vessel rupture, but with aneurysm rupture (40, 41). Intracerebral aneurysms are also prevalent in SCD patients (25, 41), and tortuosity and ectasia are well-documented in humans and animal models (42–45). Whilst aneurysms are not significantly associated with collateral vessel formation (46) they do appear to form in the context of progressive vasculopathy, with a majority of patients with aneurysms having more than one (47). In a recent clinical case review of children with SCD, five of seven patients with overt hemorrhagic stroke and/or aneurysm presented with evidence of overt ischemic stroke and/or SCI (48). These findings may indicate concurrent development of pathology underlying both ischemia and hemorrhage (49), with shared underlying mechanisms (50). Further support for this notion comes from the identification of a number of common, albeit non-specific, risk factors for both ischemic and hemorrhagic stroke, including anemia, chest syndrome, hypertension, and previous infarction (15, 51).

## Cognitive Difficulties

Overt stroke was originally identified as the primary cause of cognitive impairment in SCD (52). However, subsequent work has indicated that, whilst overt stroke and SCI are typically associated with the greatest impairment, cognitive difficulties may be common even in patients with no observable MRI abnormality (53, 54), manifesting as poorer school-readiness during the preschool years (55, 56), academic difficulties during childhood through adolescence (57–59), and employment difficulties during adulthood (60).

Already in infancy, up to 50% of patients show delay in early markers of cognition and expressive language (61). Throughout development, patients continue to be at risk of impairment across a range of domains including executive function, memory, and processing speed (27, 62–67). Although several authors have highlighted the need to consider SCD in the framework of a neurodevelopmental disorder (68), there have been no comprehensive longitudinal studies modeling raw cognitive trajectories over time. The extent to which later cognitive impairment is causally related to earlier developmental delay, and/or previous/ongoing pathophysiological processes, therefore remains unclear.

## Macro- and Microstructural Brain Alterations

Quantitative MRI studies have indicated that the total extent of cerebral tissue injury may go beyond overt stroke, SCI, and large vessel disease in SCD. There have been reports of reduced cortical and subcortical gray matter volumes (69–72) as well as reduced subcortical white matter volumes (73–75). Abnormal patterns of brain maturation have also been described (76–78). Diffusion imaging studies have further revealed significant reductions in white matter integrity, with watershed regions of the centrum-semiovale consistently affected in SCI patients well as in those without MRI-defined lesions (79–83).

Several studies have provided evidence that volumetric and structural integrity alterations contribute to cognitive impairment in patients with and without SCI. Lower gray matter volumes have been associated with worse performance IQ in adults (72), with decline in FSIQ in children (84), and with memory impairment in mice (85). Moreover, decreases in white matter density (75) and reductions in white matter integrity (86), have been associated with worse performance on tests of processing speed, irrespective of presence of SCI. It is therefore possible that cerebrovascular disease represents only the “tip of the iceberg” in terms of functionally significant cerebral tissue injury in SCD.

## Mechanisms of Neurological Morbidity

Although the incidence and impact of neurological complications in SCD are well-described, the underlying mechanisms remain poorly understood. As a result, current treatment strategies are inadequate, with many patients continuing to suffer progressive vasculopathy and/or ischemia despite being on gold-standard transfusion regimes (38, 87). Co-existing and interdependent pathophysiological processes pose significant challenges to understanding the individual impact of each in SCD. Systems and network approaches, which focus on the relationship between processes, have not been comprehensively applied, but may be useful in combination with reductionist approaches in developing an understanding of the complex pathophysiology (88).

**Figure 2:**
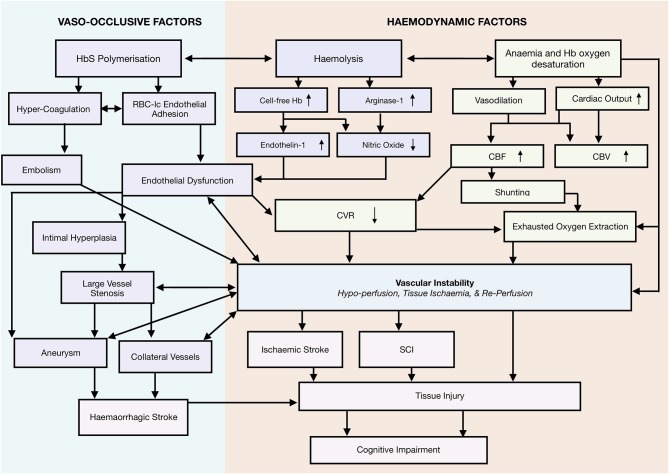
Systems biology framework. Proposed Model of neurological risk emphasizing role for vascular instability. Highlighting several potential mutually enforcing pathways. Different colors used to differentiate different mechanistic pathways, and to distinguish them from outcomes.

Taking a systems-biology approach to neurological complications, we propose a novel framework that emphasizes a role for vascular instability as a linking pathophysiological explanation for the various implicated mechanisms, including vaso-occlusion, hypercoagulability, thrombosis, hemolytic anemia, and hypoxia, as well as the interactions between them (Figure 2). According to this tentative framework, vascular instability is in part a result of operating at the limits of hemodynamic compensation for these physiological mechanisms. In this state, tissue oxygen supply is fragile and easily perturbed by relatively minor changes in clinical condition, with the potential for overt stroke, SCI, and/or micro-structural brain injury if metabolic demand exceeds tissue oxygenation. In the following sections, we review frequently implicated mechanisms, and demonstrate how the proposed framework is able to integrate them with the most current evidence in the field.

## Vaso-occlusion and Cerebral Small Vessel Disease

Vaso-occlusion was originally proposed to cause progressive vasculopathy and stroke, with erythrocyte adhesion, sickling, sludging, and congestion in small arterioles and venules. When it became clear that patients with SCD and overt stroke had large vessel disease, the suggestion that this process involved the vaso-vasorum network of feeders of large-vessels, gained traction as an explanatory mechanism for both macro- and micro-circulatory pathology (34). However, with the later discovery that large intracranial vessels lack a vaso-vasorum, the notion that vaso-occlusion alone is the proximate cause of macro-circulatory pathology has been challenged (50). Erythrocyte adhesion and congestion in post-capillary venules, with backward propagation and potential vascular pruning [i.e., regression; (89)], nevertheless remain an influential model of micro-circulatory pathology or “cerebral small-vessel disease” [CSVD; (90–93)].

Despite a lack of histological evidence, SCI, structural abnormalities, and cognitive impairment are often described as manifestations of CSVD, secondary to vaso-occlusive pathophysiology in SCD (94, 95). CSVD is typically regarded as a “whole-brain” disease, encompassing not only white-matter hyperintensities, but also other diffuse pathologies including silent micro-hemorrhages, white matter hyperintensities similar to SCI but in non-SCD populations, lacunar infarcts, and prominent perivascular spaces (96). However, the scanty available data do not suggest a high prevalence of silent microhemorrhages in children and young adults with SCD (97, 98). Moreover, whilst susceptibility-weighted MRI (SWI) of the brain has revealed patterns consistent with venular rarefaction in SCD patients (98), which may indicate vascular pruning (89, 93), concerns have been voiced about the potentially confounding effects of decreased hemoglobin and increased cerebral blood flow on SWI signal (98). Moreover, erythrocyte congestion, CSVD, and/or vascular pruning cannot alone account for the disproportionate vulnerability to injury of the deep watershed white matter regions in SCD (30, 31).

## Endothelial Dysfunction

Models have also been proposed in which neurological complications are thought to occur as a result of downstream ischemia from progressive large-vessel vasculopathy (16). It has been postulated that endothelial damage, exacerbated by inflammation, hyper-coagulation, and erythrocyte-leukocyte adhesion to the endothelium, may play a cardinal role in progressive large-vessel vasculopathy and perhaps also in CSVD and capillary pruning (16, 51, 93). There is indirect clinical evidence in support, with studies reporting associations between risk of cerebral infarction and leukocyte count (99), leukocyte expression of L-selectin (100), and endothelial expression of VCAM1 variant(-1594) (101).

According to one model of large-vessel vasculopathy (50), endothelial dysfunction may either lead to a reparative response involving intimal thickening and smooth muscle-cell proliferation or to fragmentation of the elastic lamina, with the former resulting in vessel narrowing and the development of stenosis, and the latter in vessel wall dilation and aneurysm formation. It has been suggested that local rheology, shear-stress, and/or tissue characteristics may determine whether endothelial injury leads to focal narrowing or dilation (48). Although the precise mechanisms are not well-defined, this model is able to account for cases in which regional ischemic and hemorrhagic pathology develop concurrently (48).

However, whilst associated with an increased risk of ischemic events, there is evidence that intracranial vasculopathy alone is neither necessary, nor sufficient, for the development of overt ischemic stroke, SCI, reduced integrity, or cognitive impairment in SCD. Neuropathological (49), angiographic (34, 102), and MRA/I (30, 38, 71, 82, 103, 104) studies have consistently described cases of overt ischemic stroke and SCI both in the presence and absence of observable intracranial vasculopathy. Conversely there have been reports of intracranial vasculopathy in the presence and absence of SCI and overt stroke (104–106).

For example, in a large trial (*n* = 516) in which patients with prior overt stroke or abnormal transcranial-doppler (TCD) screening results were excluded, 84% of children with SCI showed no MRA evidence of intracranial vasculopathy at baseline, and 36% of those with MRA defined vasculopathy showed no evidence of SCI (104). At exit, only 1 of 15 patients with SCI recurrence had baseline vasculopathy (107). Similarly, in a medium-sized trial (*n* = 150) in SCD children with prior overt stroke, there was no consistent pattern of intracranial vasculopathy associated with SCI, and there were no consistent hematological biomarkers for SCI or vasculopathy (87). Reduced cortical thickness (71), sub-cortical volumes (69), white-matter integrity, and cognitive performance (82, 86) are also well-documented in patients without MRI-defined lesions or MRA-defined vasculopathy. Whilst these studies are plagued by highly heterogeneous samples and use of inconsistent vasculopathy definitions (32), these and other findings have nevertheless encouraged authors to explore alternative etiologies for neurological morbidity in SCD (108).

## Hypercoagulability and Embolic Events

There is indirect evidence that cerebral embolic events occur in SCD patients, including reports of associations between overt ischemic stroke and thromboemboli (109, 110) as well as of fat-embolism syndrome from bone-marrow necrosis (111–114). Although comprehensive prevalence data are lacking, shunting at intra-pulmonary or intra-cardiac (e.g., through a PFO; patent foramen ovale) level and paradoxical embolism may also be more common in children (33, 115, 116) and adult (117) SCD patients and may be associated with cerebral infarction (118). Although there are few data in SCD, PFO is an established risk-factor for overt stroke in the general population (119–121).

Hypercoagulability may pre-dispose to cerebral thromboembolism and is also a feature of SCD. Activation of the coagulation cascade and fibrinolysis are favored (122) and there is a high risk of venous thromboembolism (123). Risk factors may include genetic predisposition (124), inflammation (122), and splenectomy (125). Phosphotidylserine exposure on red cells and microparticles may play a role, related in part to acquired protein S deficiency (126). Whole blood thrombin generation is increased in SCD, while plasma thrombin generation is decreased, suggesting a cellular component, although this does not appear to be related to phosphotidylserine exposure (126). There is cross-sectional evidence indicating that hypercoagulability may contribute to risk of overt stroke and SCI in SCD patients (127, 128). Proteomic analyses have revealed associations between SCI and the prothrombotic proteins α2-antiplasmin, fibrinogen-γ chain and thrombospondin-4 which are considered to predispose to hypercoagulability (124). Although findings have been mixed, several studies have also shown lower serum levels of coagulation markers [e.g., D-Dimer, Von Willebrand factor, TAT complex; (129)] and lower thrombin generation (130) in SCD children deemed at low risk of overt stroke on the basis of transcranial doppler (TCD) velocities (see page 10). In addition, poor splenic function is associated with SCI (99), although the link to hypercoagulability has not been made for SCD as it has for thalassemia (131, 132).

Upregulation of platelets may exacerbate the hypercoagulable as well as the proinflammatory state associated with SCD. Platelets may also promote endothelial activation and erythrocyte adhesion by stimulating several major endothelial adhesion molecules, including vascular adhesion molecule (VCAM-1) (133) and by forming an increased number of platelet-erythrocyte (134, 135), platelet-monocyte (136) and platelet-neutrophil aggregates (136). Although the underlying mechanisms are unclear, there is cross-sectional evidence that patients with SCI or overt ischemic stroke have higher mean platelet values than patients without lesions on MRI (137). Also, thrombocytosis (platelets > 500^*^10^9^/L) is associated with cognitive impairment across multiple domains in children with SCD (138), and elevated levels of erythrocyte and platelet derived microparticles have been described in those with a history of overt stroke (139). Whilst there is also evidence that higher mean platelet volume is associated with a global increase in white matter volume in SCD patients, further work is required to determine whether this is adaptive or a reflection of edema (74). Given these data, there is a good case for further investigation into the relationships between platelet activation, hypercoagulability, and neurological complications in SCD. Mechanisms could include embolism through a cardiac or pulmonary shunt from the systemic venous circulation e.g., in the pelvis or the limbs, as well as local thrombosis.

## Hemodynamic Compromise

### Watershed Vulnerability

Whilst vaso-occlusive, thrombotic, and/or embolic events may contribute to some ischemic insults in SCD, several authors have argued that the high density of overt and silent infarction and microstructural abnormalities in watershed regions may point to hemodynamic compromise or “brain drain” as a more common contributor (32, 140). Historically, in non-SCD patients, watershed infarcts have been associated with hemodynamic causes, and are sometimes referred to as hemodynamic strokes (141, 142). As the watershed regions lie at the end junctions between adjacent arterial territories, vascular supply is inherently low. Much as the last field on a farm is the area with the least supply of water and therefore the most vulnerable to a reduction in flow, the watershed regions of the brain are believed to be the most vulnerable to a reduction in perfusion (143).

### Vascular Physiology

Despite only accounting for 2% of total body weight, the brain has the highest metabolic requirements of any organ, consuming a disproportionate 20% of the body's total oxygen supply. In children, brain oxygen consumption is even higher, reaching 50% during the first decade of life (144). As reflected by these high demands, a baseline cerebral metabolic rate of oxygen utilization (CMRO_2_) is required to maintain tissue viability (145).

CMRO_2_ is defined as the product of arterial oxygen content (CaO_2_), rate of blood delivery (CBF; Cerebral blood flow), and the percentage of oxygen extracted by the tissue (Oxygen extraction fraction; OEF).

The following equations, derived from the Fick principle show their relationship;

$$\begin{array}{l} \;{\text{CaO}}_{2}=({\text{Hemoglobin}}^{*}\text{1}.{\text{34}}^{*}{\text{SaO}}_{2})+(\text{0}.{\text{003}}^{*}\;{\text{paO}}_{2})\\ \text{Oxygen Delivery}={\text{CaO}}_{2}^{*}\text{ CBF}\\ \text{ OEF}=({\text{CaO}}_{2}-{\text{CvO}}_{2})/{\text{CaO}}_{2}\\ \;{\text{CMRO}}_{2}={\text{CaO}}_{2}^{*}{\text{CBF}}^{*}\text{ OEF} \end{array}$$

Where 1.34 is the oxygen affinity of normal hemoglobin type A, paO_2_ is the partial pressure of oxygen in arterial blood, SaO_2_ is the ratio of oxygenated hemoglobin to the sum of oxygenated and deoxygenated hemoglobin in arterial blood, and CvO_2_ is venous oxygen content (C_v_O_2_) defined similarly to CaO_2_, but with metrics drawn from venous rather than arterial blood.

In normal vascular physiology, CBF is closely coupled to baseline CMRO_2_, leading to globally uniform OEF (146, 147). By arteriolar dilatation, CBF increases in response to increased metabolic demand related to function, e.g., movement of a limb or response to a visual stimulus. Under conditions in which oxygen delivery is decreased [e.g., hypoxia; (148), carotid artery occlusion; (146)] or CMRO_2_ is increased beyond normal functional demands [e.g., pyrexia or seizures; (149)] the brain is able to fall back on two reserves; a cerebrovascular dilatory reserve (CVR) and a metabolic reserve. CVR reflects the capacity of smooth muscles to alter vessel caliber in response to fluctuations in arterial blood gases such as carbon dioxide and oxygen (150). The arterioles respond to changing carbon dioxide tension with a positive linear response across the physiological range but flattening at the extremes (151). Although the underlying mechanisms are less well-understood, the metabolic reserve reflects the capacity of the brain to augment CMRO_2_ via increases in OEF, which may potentially involve changes in effective oxygen diffusibility (152).

In models of hemodynamic stroke there is a disproportionate drop in oxygen delivery relative to baseline CMRO_2_, and an exhaustion of vascular reserve mechanisms (141)_._ Within the first 48 h of an ischemic insult, a state of hemodynamic compromise known as “misery perfusion” is often observed, involving reductions in regional CBF that are accompanied by increases in regional OEF. Regional OEF increases may serve to maintain CMRO_2_ up to a point, beyond which tissue injury may ensue (146, 153–155).

There have been reports of hemodynamic changes consistent with a similar model of hemodynamic compromise in patients with SCD, including altered CaO_2_, CBF (156–161), CVR (162–164), and OEF (165–167). Whilst vaso-occlusion, vasculopathy, and emboli are all flow-restricting phenomena that may contribute to hemodynamic compromise, some hemodynamic changes may represent compensatory responses to physiological stressors associated with SCD pathophysiology (140), including anemia and hypoxia. In the following subsections, we consider research on aspects of CMRO_2_ in SCD in turn.

### Arterial Oxygen Content (CaO_2_)

In patients with SCD, hemolytic-anemia leads to chronic hemoglobin-driven reductions in CaO_2_ (6). CaO_2_ may, however, be further reduced in these patients due to acute, intermittent, and/or chronic daytime, nocturnal, and/or exercise-induced oxyhemoglobin desaturation. Daytime oxyhemoglobin desaturation, when defined by pulse oximetry as SpO_2_ < 96%, may affect between 30 and 50% of steady-state patients (168–173).

Although a well-described phenomenon, there is no consensus on cause, definition, or treatment of oxyhemoglobin desaturation in SCD (174). Proposed mechanisms include phenomena often considered “hypoxemic” such as abnormal HbS oxygen affinity, elevated levels of dyshemoglobins, and pulse oximeter calibration for HbA rather than HbS (175, 176). “Hypoxic” phenomena, including obstructive and restrictive lung disease, sleep disordered breathing, and shunting, have also been proposed to play a role (176, 177).

The affinity of hemoglobin for oxygen is a fundamental determinant of the oxygen-carrying capacity of blood and is altered in patients with SCD. HbS polymerization has long been known to reduce oxygen affinity, causing a right shift of the oxyhemoglobin dissociation curve [ODC; (178–181)]. Although there is significant heterogeneity, the pO_2_ at which hemoglobin is 50% saturated (P50) is increased in a majority of SCD patients, meaning that hemoglobin oxygen saturation for any given pO_2_ is lower (171, 182–185). Whilst this right shift of the ODC is seen in many anemia's, and facilitates unloading of oxygen from blood to tissue (see section below on OEF), it inhibits oxygen loading at the lungs, which may promote oxyhemoglobin desaturation in SCD (186).

Studies using near-infrared spectrophotometry have provided evidence that cerebral oxyhemoglobin tissue desaturation is common and can be severe in steady-state SCD patients (187–189). However, oxygen carrying capacity appears to only partially explain cerebral desaturation, with CaO_2_, age, and male gender together accounting for 40% of the variance (188).

### Cerebral Blood Flow, Cerebrovascular Reserve, and Cerebral Autoregulation

Cerebral tissue oxygenation is dependent not only upon oxygen availability and the blood's oxygen carrying capacity, but also on tissue perfusion. Whilst CaO_2_ is chronically decreased in SCD patients, studies have consistently reported compensatory vessel dilation (190), leading to increases in global CBF and CBV (157, 166, 191, 192), which appear to maintain oxygen delivery and metabolism when averaged globally (157, 167).

However, in patients with SCD, compensatory increases in global CBF are associated with reduced CVR (70, 162–164, 193), with the white matter also exhibiting disproportionate delays in CVR response times (194). There is evidence that a majority of patients may approach the upper limit of dilatory capacity, and that a quarter may also exhibit negative reactivity or “steal” (193). Steal refers to blood being “stolen” from one cerebral region and given to another, and occurs when a pressure gradient exists between regions, such as when one region is maximally dilated and unable to respond to a vasodilatory stimulus [e.g., hypercapnic challenge; (195)]. In these instances, blood may be redistributed from regions unable to dilate to regions that are able to. Theoretically, therefore, in a parallel vascular system where there is CVR exhaustion, an increase in perfusion in one region can lead to a relative decrease in perfusion in another.

Studies in healthy populations suggest that some brain regions are more vulnerable to CVR exhaustion and steal than others. CVR appears to be greater in gray matter than white matter (196, 197), as well as in phylogenetically older than phylogenetically younger gray-matter regions of the brain (198, 199), which may be related to the relatively greater vascularization in gray matter regions that perform essential homeostatic functions (198). There is also evidence that watershed white matter regions are disproportionately at risk of steal in young healthy populations during hypercapnia (196), suggesting that these regions may be continuously compensating for low perfusion pressure. Exhausted CVR, alone or in combination with steal, may thus render the watershed white matter regions disproportionately vulnerable to ischemia in settings where there is increased metabolic demand (e.g., infection, pyrexia, seizures) or an acute drop in CaO_2_ [e.g., acute chest syndrome with acute anemia and hypoxia; (33)], which are common in SCD.

Whilst several MRI studies suggest that global white matter CBF is on average elevated in “steady-state” SCD patients (158, 166, 200), the elevation is lower than that observed for gray matter, and may therefore be insufficient to maintain oxygen delivery regionally. Results from a more recent study are consistent with this notion, and suggest that global white-matter oxygen delivery is significantly reduced in “steady-state” SCD patients without MRA defined vasculopathy compared to controls (201). Using a rigorous partial volume correction, the authors found significantly elevated global gray-matter CBF in patients, but no differences in global white-matter CBF, indicating inadequate compensatory vasodilation in white-matter. Importantly, through t-score maps, the authors showed the reduction in white matter oxygen delivery to be disproportionate in watershed regions vulnerable to SCI and reduced integrity, going beyond that expected due to anatomical constraints and the watershed effect alone (201). These findings suggest that watershed regions are hypo-perfused in SCD patients, and highlight the need for future studies to consider regional perfusion characteristics alongside global averages. In line with this, Positron Emission Tomography [PET; (161, 192, 202)], Single Photon Emission Computed Tomography [SPECT; (203–205)], Xenon-Computed Tomography [CT; (206)] and MRI studies (158, 160, 201) have also reported regions of hypo-metabolism and/or hypo-perfusion in patients with SCD.

The etiology of regional hypo-perfusion in SCD is unclear. In the absence of longitudinal data, it is impossible to determine whether injury in these regions occurs secondary to hypo-perfusion, or whether hypo-perfusion is secondary to the lower metabolic requirements of injured tissue. Given that CBF and CMRO_2_ are closely coupled, it is possible to speculate that injured regions have lower CMRO_2_, resulting in hypo-perfusion. However, SCI-burden was relatively low in the Chai et al. (201) sample, with only half of patients showing small lesions. Moreover, there is evidence that even in “steady-state” patients without SCI, PET may find regions of hypo-metabolism and hypo-perfusion (161). Whilst CVR-exhaustion and vascular steal secondary to compensatory increases in gray matter CBF is another plausible explanation, it is also possible that there is a broader vulnerability of vasculature regulation in SCD.

Cerebral autoregulation (CA) refers to the ability of the brain to maintain relatively constant CBF over a broad range of cerebral perfusion pressures (CPP), and is thought to involve a complex interplay of autonomic, myogenic, and neuronal mechanisms (207).

Cerebral perfusion pressure (CPP) is defined as either:

$$\begin{array}{l} \text{CPP}=\text{MAP- ICP}\\ \text{ or}\\ \text{CPP}=\text{MAP- JVP} \end{array}$$

Where MAP is the mean arterial pressure and JVP is the jugular venous pressure.

If blood pressure decreases or increases, CA maintains constant CBF across the autoregulatory range which varies with age and a variety of conditions. Below this range, CBF falls with decreasing CPP, risking ischemia, particularly in the watershed regions. Above this range, CBF rises with increasing CPP, with the risk of edema, particularly in the posterior circulation. There is some evidence indicating impaired CA in SCD patients, with one study showing that patients have a reduced capacity to buffer the transfer of blood pressure surges to the cerebral tissue (208). Whilst CA has traditionally been treated as separate from CVR, both are mechanisms deployed to ensure CBF-CMRO_2_ coupling in the face of changing physiological conditions, and there are persuasive data indicating synergism and interdependence between them (150, 209, 210). For example, progressive hypotension appears to blunt and abolish the CBF response to hypo and hypercapnia (151, 211), and hypoxia and hypercapnia appear to reduce the ability of the brain to defend against changes in perfusion pressure as well (212, 213), suggesting that CVR and CA may rely on the same underlying flow reserve.

The role of reduced CPP secondary to intra/or extracranial vasculopathy (24, 35), diastolic dysfunction (214), relative systemic hypotension (215), and/or embolism, has received relatively little attention in SCD, but any effect may be compounded by CVR exhaustion. Some SCD patients may thus face a “quadruple jeopardy” of reduced CaO_2_, systemic hypotension, CBF restricting stenosis/emboli, and exhausted CVR (140). It is unclear whether low flow conditions are further exacerbated by increases in JVP secondary to erythrocyte congestion in post-capillary venules, and/or increases in ICP secondary to acute drops in CaO_2_ and cerebral oedema [e.g., in acute hypertension; (216) or hypoxia; (217)]. However, it is possible that critical closing pressure, the CPP at which vessels collapse and close completely, is reached during acute illness with relatively small increases in either ICP or JVP or reductions in MAP.

Both CVR and CA must necessarily rely on the same underlying capacity for cerebral vessels to regulate resistance (150), a capacity which is modulated by local metabolites, RBC chemistry, the autonomic nervous system, and blood rheology, all of which are abnormal in SCD (218). Vessel caliber is ultimately dependent on the balance between the myriad of vaso-constricting and vaso-dilating agents derived from the endothelium, neuronal innervations, and physical factors such as shear and stretch (219). Evidence from forearm and renal studies suggests that the vaso-active balance is inherently vulnerable in SCD patients, with concomitant upregulation and exhaustion of vaso-constricting and vasodilating agents (220). For example, low nitric oxide (NO) bioavailability occurs secondary to hemolysis in patients with SCD, and given that NO is a powerful vasodilator that also inhibits the vaso-constrictive effect of endothelin-1, this may increase reliance on other agents and tip the balance in favor of vaso-constriction once alternative agents are exhausted (91, 221).

Although it is unclear how this plays out in the cerebral circulation in SCD, and the molecular mechanisms underlying CVR and CA remain the subject of much debate (150, 222), studies in animals and humans suggest that endothelial NO may play a role in moderating CVR as well as in extending the lower limit of CA (223, 224). In endothelial nitric oxide synthase knockout mice, for example, there is a substantial rightward shift of the CA curve at low perfusion pressures (225). Whilst a right shifted CA may protect the brain from brain-barrier disruption secondary to hyper-perfusion, it may also mean that a higher perfusion pressure is required to prevent hypo-perfusion.

Of note, impaired CA (226–228) and reduced CVR (229, 230), have also been observed following sympathetic stimulation in animals and humans. There is evidence for autonomic nervous system dysfunction in SCD, with enhanced sympathetically mediated vasoconstriction reflexes (218, 231–234), which theoretically, could compound any effect of reduced NO. Although there are no data comparing CVR, CA, and the interaction between them in SCD, a vulnerability in the availability of regulatory agents, either alone or in combination with autonomic nervous system dysfunction, may mean that normal CVR and CA ranges are right-shifted and/or narrower with loss of the plateau. Coupled with the inherent anatomical vulnerability of watershed white matter regions, reduced and/or altered regulatory capacity may further predispose SCD patients to hypoperfusion and/or oxygen supply-demand mismatch in these regions.

Theoretically, in patients with higher hematocrit, either naturally or as a result of transfusion, the increased viscosity of blood containing HbS could exacerbate hypo-perfusion in low-shear watershed regions (51, 235, 236). There is *in-vitro* evidence, including in patients with SCD, suggesting that a lower hematocrit to viscosity ratio (HVR) measured at high shear rate is associated with poorer cerebral oxyhemoglobin saturation as measured by NIRS (237). However, HVR is a measure that confounds CaO_2_ and viscosity, meaning that it could be low secondary to either low CaO_2_ or high viscosity. Moreover, studies in animals using high and low viscosity replacement fluids (238) as well as in humans with other anemia's, polycythemia, and paraproteinemia (148, 239), suggest that once any differences in CaO_2_ are accounted for, the impact of viscosity on global CBF is negligible. These findings have been replicated in other populations with normal vascular function and hematocrit during isovolumic conditions (240). This apparent contradiction of Poiseuille's law may relate to the physiological conditions of the cerebral vasculature, with turbulent flow, non-Newtonian fluid, and atuoregulation of vessel caliber (224). However, if vessels are less able to dilate in SCD patients, either due to CVR exhaustion secondary to reduced CaO_2_ and/or a broader vulnerability in regulatory capacity, the ability to compensate for increases in viscosity and/or reductions in deformability may be reduced. Whilst there is some data indicating no independent effect of blood viscosity on global CBF in patients with SCD (157), further work is required to examine the effects of viscosity and deformability in both low and high shear regions of the brain.

### Mutually Enforcing Pathways

Of note, hypo-perfusion reduces shear-stress, and there is evidence that endothelial cells exposed to low-shear conditions show sustained activation of adhesion molecules, tissue factors, and inflammatory agents, as well as decreased production of nitric oxide (241, 242). Hypo-perfusion may also increase the risk of thrombus formation secondary to platelet-aggregation (243). Interestingly, murine studies have demonstrated that pre-conditioning via prior exposure to ischemia can be neuroprotective by reprogramming the genetic response to ischemia, with adaptations including the suppression of thrombus formation (244). Presence or absence, or even the degree and timing, of pre-conditioning may be relevant in determining the nature of acute neurological presentations in SCD where patients are at risk of chronic sustained and intermittent exposure to hypoxia (245).

There is also evidence that high and turbulent shear-stress, which may occur secondary to hyper-perfusion and reduced CaO_2_, can induce angiogenesis and vascular remodeling (241, 246, 247). Hypoxic exposure may additionally promote angiogenesis through several non-mechanic endothelial pathways (248), although these may be perturbed in SCD. Nevertheless, reports indicate that patients with SCD display a heightened “angiogenetic tone,” with elevated levels of proangiogenic growth factors, which in combination with endothelial dysfunction, could contribute to vasculopathy (4). Taken together, these findings illustrate how mutually enforcing pathophysiological processes may be at play, and suggest that, depending on the extent of any pre-conditioning (e.g., via prior exposure to hypoxia), both global hyperperfusion and regional hypoperfusion could in turn exacerbate erythrocyte-leukocyte adhesion, hypercoagulation, endothelial dysfunction, and vasculopathy (249).

### Oxygen Extraction

Reports that OEF is abnormal (165, 167), particularly in watershed regions prone to SCI (166), are further indicative of hemodynamic compromise and regional vulnerability in SCD patients (250). There is evidence that changes in global OEF are associated with increases in global CBF, but that only changes in OEF are related to higher levels of clinico-radiological impairment, defined as moderate stenosis >50% in any major vessel, prior overt stroke or SCI, and/or chronic debilitating pain (167). Although there is controversy as to whether oxygen extraction is higher (166, 167, 251) or lower (165, 252), both patterns would be consistent with on-going metabolic stress, with higher or lower OEF potentially either reflecting compensation for, or exacerbation of, hemodynamic compromise.

These paradoxical findings may be explained in the context of preliminary reports of venous hyperintensities on arterial-spin labeling MRI, consistent with arterio-venous shunting (253). One theory, named the “functional shunting hypothesis” (254), postulates that regional shunting pathophysiology, coupled with compensatory increases in global CBF and reductions in arterial transit times and CVR, lead to regions of impaired oxygen unloading and diffusion in SCD, reflected by regional reductions in OEF. In these instances, tissue oxygen delivery may be compromised even though differences between arterial and venous saturation are small. Such shunting could be compensatory in terms of minimizing HbS polymerization and/or metabolic demand [e.g., hibernation; (165)], but could also be a dysfunctional consequence of macrocirculatory hyperperfusion in the setting of reduced and/or shifted CA.

The functional shunting hypothesis has been challenged, however, with one study finding no relationship between venous hyperintensities and global OEF (253, 255). Part of this controversy stems from the need for calibration models when using oxygen-sensitive MRI techniques. In SCD patients, T2 relaxation under spin tagging (TRUST) can yield diametrically opposing results depending on data calibration model [HbA vs. HbS calibration; (165, 256)], and there is no consensus on model validity (255). As a result, global CMRO_2_ in patients with SCD has been reported to be higher (166, 257, 258), lower (165, 252), and similar (167) to that of controls.

Reports of higher global OEF are broadly consistent with a previous PET study (192) and have been established using two different MRI methods [asymmetric spin echo—(166); TRUST with HbA calibration–(167, 258)]. However, both depend on broad assumptions that may not be valid in SCD patients (165, 259), as demonstrated by a recent study employing a novel susceptibility-based technique, where venous oxygen saturation was found to be elevated in SCD patients, consistent with lower global OEF (259). Further complicating matters, it is possible that there are regional differences in OEF and transit times, or thresholds beyond which increases in CBF begin to impair oxygen unloading. Whilst further work is required to determine whether OEF is higher, lower, or spatially heterogenous in SCD patients, the available data are nevertheless indicative of OEF exhaustion and/or insufficiency, consistent with hemodynamic compromise, and likely exacerbated by hypoxic and anemic exposure.

Of note, shunting pathophysiology has been described in other vascular beds in SCD, including the peripheral [Upper arm; (260)] and pulmonary (118) circulations. Whilst the similarities and differences between vascular beds of various organs have received little attention and are poorly understood, the cardiac description of a “superimposed restrictive and hyperdynamic physiology” (261), and the renal, fingertip, and skeletal muscle descriptions of a “perfusion paradox” (93, 220), are similar to the hemodynamic changes observed in the cerebral circulation, with hyper-perfusion in the macro-circulation, hypo-perfusion in watershed regions of the micro-circulation, and an exhaustion of vascular reserve mechanisms. These findings are suggestive of a state of vascular instability, in which tissue oxygen supply is fragile, and easily perturbed by fluctuations in clinical condition.

## A Systems-Biology Framework

Taken together, the reviewed mechanisms are consistent with a tentative systems-biology framework of neurological morbidity with vascular instability at its core (Figure 2). According to this tentative framework, increases in CBF, reductions in CVR, and exhausted/insufficient OEF, may act synergistically to cause vascular instability (Figure 2), a state in which risk of regional hypoperfusion, ischemia, re-perfusion, and the associated inflammatory milieu are high. These factors may contribute either alone, or in combination with acute drops in CaO_2_, vasculopathy, erythrocyte congestion, and/or thrombo-emboli to perturb tissue oxygenation, leading to overt stroke, SCI, or microstructural tissue injury (e.g., reduced integrity). In this tentative framework, it is differences in the severity, duration, and precise location of a hypoxic-ischemic or hemorrhagic insult, that determine structural and functional tissue outcome.

Importantly, vascular instability provides a linking pathophysiological explanation for the various implicated processes, including vaso-occlusive, coagulative, thrombotic, hypoxic, and hemolytic phenomena, as well as the interactions between them. The framework is consistent with a previous systems-biology model of systemic vasculopathy in SCD, in which ischemia-re-perfusion injury and inflammation are emphasized, along with multiple overlapping and mutually-enforcing mechanistic pathways (88). The current neurological model similarly attempts to provide a parsimonious account of neurological risk and morbidity, in which multiple potential pathways are highlighted, but the most proximate mechanism is emphasized. Below, we consider evidence consistent with the framework.

### Evidence for Links With Neurological Morbidity

There are many strands of indirect clinical evidence broadly consistent with a role for vascular instability in neurological morbidity, with decreased hemoglobin and peripheral oxygen saturation, components of CaO_2_, consistently associated with overt stroke (11, 262, 263), SCI (20, 23, 24, 99, 264), reduced white matter volume and tissue integrity (81, 82), and cognitive impairment (86, 265–267). Several case-series have highlighted acute chest syndrome in patients presenting with overt ischemic stroke in the absence of intracranial large-vessel vasculopathy (11, 103, 268), which may indicate a role for reduced oxygen delivery and hemodynamic failure (269).

Moreover, TCD, which captures the time averaged mean of the maximum velocity of blood, can be high as a compensatory mechanism for reduced CaO_2_ (270, 271) rather than vessel narrowing, and there is evidence that up to 79% of SCD children with high TCD have either no stenosis or <25% stenosis (272). In an analysis of the STOP trial data, only 2 out of 6 high TCD patients who went on to have a stroke showed evidence of intra-cranial vasculopathy (273). Although extra-cranial vasculopathy may not have been excluded, these findings are consistent with the notion that hemodynamic factors, e.g., reduced CVR associated with high CBF, may be more pertinent to the etiology of overt stroke than vasculopathy alone.

According to one seminal model of hemodynamic stroke in non-SCD patients, transition from misery perfusion to ischemic stroke is a result of perfusion pressure dropping to such an extent that CMRO_2_ is no longer maintainable by increases in OEF (146). Whilst there are a number of PET studies in support (274, 275), isolated reports of favorable tissue outcome following misery perfusion, termed the “ischemic penumbra,” including in one patient with SCD (276), indicate that there may be additional mechanisms involved in determining transition to observable tissue infarction (146, 277).

Although the hemodynamic underpinnings have not been investigated, a similar, albeit lower level, potentially reversible phenomenon termed “acute silent cerebral event” (ASCIE), has also been observed in SCD patients (278–282). In a prospective case-series, 18% of SCD patients and 7% of non-SCD patients presenting with acute anemia (hemoglobin <5 g/dl and >30% lower than steady state) secondary to infection, acute chest syndrome, and/or fever, showed lesions consistent with ischemia on DWI, termed ASCIE (279). On follow-up MRI, a majority, but not all, patients showed evidence of SCI corresponding to the original ASCIE. In 75% of the SCD patients presenting with ASCIE, there was no evidence of vasculopathy. A more recent multi-center trial established that ASCIE were detectable and prevalent also in “steady-state” SCD patients undergoing MRI screening, with an estimated 10 times greater incidence of ASCIE compared to SCI [47.3 vs. 4.8 per 100 patient years; (282)].

The temporal association of ASCIE with acute anemia, along with the observed transition of some ASCIE to SCI, are consistent with, but do not establish, a role for reduced oxygen delivery in SCI. Given that not all ASCIE progress to permanent lesions (i.e., SCI), these findings may suggest that additional hemodynamic, vaso-occlusive, inflammatory and/or pre-conditioning mechanisms are involved in determining transition from ASCIE to observable tissue infarction.

**Figure 3:**
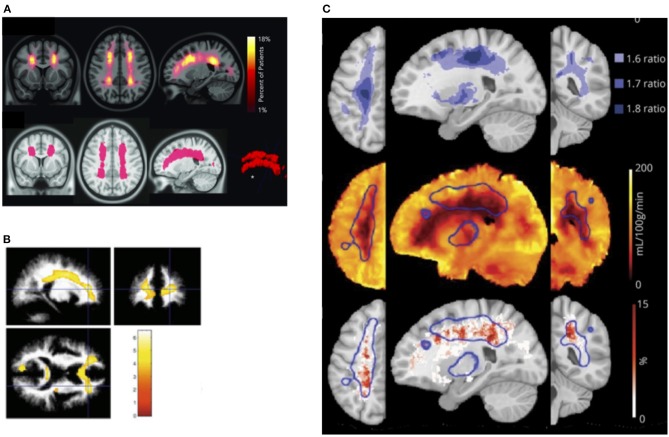
Watershed vulnerability. Results from collection of studies illustrating watershed vulnerability in SCD. **(A)** Top: SCI density map from 286 SCD children. Bottom: region encompassing 5.6% of brain volume in which 90% of SCI were confined [from Ford et al. (31)]. **(B)** Regions in which 20 SCD children without SCI demonstrated reduced white-matter density compared to 31 controls [from Baldeweg et al. (73)]. **(C)** *Top:* Ratiometric maps showing regions of elevated OEF derived from the ratio of SCD (*n* = 36) to control (*n* = 20) OEF values. **(C)** *Middle:* Region of high OEF (threshold 1.6, outlined in blue) overlaid on the average CBF map from the SCD cohort. **(C)** *Bottom:* Region of elevated OEF overlaid on SCI density map created from an independent cohort of 23 participants with SCD [from Fields et al. (166)].

It is unclear what determines this tipping point in patients with SCD, but in prospective studies of non-SCD patients with carotid occlusion, risk of infarction is highest in patients with both increased OEF and CBV, the former indicative of misery perfusion, and the latter potentially of vasodilation and exhausted CVR (283, 284). These findings are consistent with models of hemodynamic stroke and tissue ischemia in which regional reductions in oxygen delivery (201) along with both exhausted CVR and OEF may play mechanistic roles. Recent SCD research indicating that regions of CBF and CMRO_2_ nadir overlap with the regions of highest SCI density (31) and highest oxygen extraction (166), provide further indirect evidence for a similar model in SCD (Figure 3). Research on these hemodynamic factors is, however, just beginning. Further work is required to establish whether concomitant measurement of CBF, OEF, and CBV/R may lead to better stratification of neurological risk than TCD in patients with SCD.

Whilst the mechanisms by which transfusion reduces risk of stroke are unknown and vigorously debated, there are some reports indicating that a reduction in vascular instability may play a role. Transfusion significantly increases CaO_2_, and has immediate hemodynamic effects, reflected by reductions in CBF (285) and TCD velocity (286). Post-transfusion reductions in OEF (191, 255) and increases in CVR (162) have also been reported. Similar hemodynamic changes in global OEF and CBF have also been observed following bone marrow transplantation (287), which is the only curative treatment option currently available for SCD. Taken together, these findings provide proof of principle that normalization of CaO_2_ and hyperemia, along with restoration of vascular reserves, may contribute to the efficacy of transfusion in reducing risk of overt stroke.

Interestingly, post-transfusion reductions in global OEF and CBF are independently associated with improvement in total hemoglobin, but not HbS fraction (191, 255, 288), which suggests that a reduction in vascular instability is primarily achieved via improvement in global oxygen delivery rather than RBC rheology, and has implications for current transfusion strategies with HbS% targets. However, given their interdependence, these effects are difficult to disentangle. In SCD, both the compensatory global increases and post-transfusion reductions in CBF are greater than would be expected from changes in hemoglobin levels alone (289), suggesting that factors beyond correction of CaO_2_ are at play.

Moreover, transfusion appears to reduce, but not completely normalize, CBF and OEF in SCD patients, with watershed zones continuing to exhibit “at-risk” regions (191). There is also evidence that OEF and CBF responses to transfusion are blunted in adult SCD patients (255). These factors could contribute to continuing risk of morbidity in some patients, and may relate to vaso-occlusive/rheological factors, endothelial dysfunction, concomitant shifts in the oxygen-dissociation curve, and/or reduced regulatory capacity. There is *in-vitro* evidence that low-shear HVR decreases following simple chronic transfusion therapy in SCD patients, indicating that despite improvement in CaO_2_, post-transfusion increases in blood viscosity may worsen oxygen delivery in low-flow regions (235, 290). However, these findings are inconsistent with the observation that “at risk” regions of elevated OEF in watershed white matter zones appear to become smaller, rather than larger, following exchange transfusion (191). A possible explanation for this apparent juxtaposition is a difference in flow mechanics following simple and exchange transfusion, with exchange transfusion significantly reducing HbS% without substantially increasing hematocrit and viscosity (291). It is also possible that post-transfusion increases in CaO_2_ and CVR somewhat restore the ability of the brain to compensate for slight increases in viscosity.

Consistent with this notion, a recent study comparing untreated, chronically exchange transfused, and hydroxyurea (HU)-treated SCD patients, a less invasive treatment strategy based on stimulation of fetal hemoglobin (HbF), found OEF to be lowest in the transfused patients (288). Whilst “at-risk” regions of elevated OEF in watershed zones were detected across all groups, they were larger in the untreated and HU-treated patients than in the transfused patients, respectively. Interestingly, global gray and white matter CBF were similar among all groups, and there were no differences in total hemoglobin or SpO_2_ between HU-treated and transfused patients, suggesting that the between-group differences in OEF are not explainable by differences in global oxygen delivery. Of note, given that imaging was conducted on the day before scheduled transfusion, and other studies have shown reductions in global CBF and OEF 24 h post-transfusion (191), these findings may suggest that the hemodynamic effects of transfusion are greater near transfusion, compared to far from transfusion, as has been demonstrated for cognitive impairment (292). Nevertheless, the apparent inferiority of HU, even when compared with “late” transfusion effects, may be accounted for by the increased affinity of hemoglobin F for oxygen (293). Whilst the authors found no independent effect of HbF% or HbS% in multivariate models, left shifts in the oxygen dissociation curve are likely to impair oxygen offloading, and may be greater following HU than following transfusion. Whilst the effect on global oxygen metabolism may be balanced by the concomitant improvement in global oxygen delivery, more work is required to establish whether this is the case also for regional oxygen delivery, particularly in view of the finding that “at-risk” regions remain.

Whether vascular instability contributes to structural delay/deterioration not visible using conventional MRI and associated cognitive impairment, is an open question (294). The increased prevalence of ASCIE compared to SCI in acutely ill as well as steady state patients is consistent with the notion of an on-going state of vascular instability, and suggests that risk of ischemic insult may be far higher than previously recognized in SCD. It is unclear whether some of these insults are radiologically reversible or lead to microstructural tissue injury not visible using conventional MRI techniques. In support of the latter possibility, there is evidence that lesion detectability increases with increasing magnet strength in SCD, with one study showing that 3T MRI fails to detect lesions that are visible at 7T (295). Also, glial fibrillary acidic protein (GFAP), a marker of acute stroke and brain trauma, is significantly upregulated and associated with performance IQ, but not verbal IQ in “steady state” SCD patients with and without SCI (296).

Correlations have also been demonstrated between reductions in CVR and cortical thinning in regions of high metabolic activity in children with SCD (70), which may suggest that reduced dilatory capacity is involved in more subtle, and widespread tissue atrophy and/or delayed maturation. This notion is further supported by a recent report demonstrating a disruption in the relationship between CVR and white-matter integrity in SCD patients (297). There is evidence that reduced integrity is more common (82), and potentially also more functionally significant than SCI alone in SCD patients (86). Case reports of deterioration in cognitive function with acute drops in CaO_2_ in SCD (33) along with studies showing correlations between TCD abnormalities and executive dysfunction (298–300) and between reduced blood-oxygenated dependent (BOLD) MRI responses to visual stimulation and intelligence (301), lend further support to a role for vascular instability in cognitive impairment.

## Conclusion and Future Directions

In summary, the pathophysiology of neurological morbidity in SCD is complex, and likely involves multiple mutually enforcing pathways, including vaso-occlusive/rheological, hemolytic, and hypoxic phenomena. Based on existing theories and accumulating evidence, we have proposed an integrative framework which emphasizes a role for vascular instability as a potential linking pathophysiological explanation. This framework brings together recent developments in the field, highlights outstanding questions, and provides mechanistic hypotheses that may guide future research.

Whilst the many strands of indirect evidence presented are broadly consistent with the framework, they do not rule out alternative and/or additional mechanisms of neurological morbidity. In order to interrogate and refine the framework, further advanced MRI studies are required. For this purpose, longitudinal measures of oxygen-metabolism would be most useful. As the framework and reviewed literature demonstrate, aspects of CMRO_2_, such as oxygen delivery and extraction ought to be considered together, both globally and regionally. Multi-modal, neurodevelopmental approaches that combine structural, diffusion, hemodynamic, and cognitive measures would also be helpful in further addressing outstanding questions.

One of the key challenges with these advanced MRI techniques remains validation in SCD patients, in whom some of the underlying assumptions may not be valid (165, 256, 259, 302–304). Comparison with current clinical gold-standard (e.g., PET for oxygen-extraction) may be useful in this regard. Employment of standardized criteria for detection of SCI and grading of vasculopathy will also be important in facilitating between-study comparisons (32, 164). Given that the vast majority of reviewed studies are based on patients with homozygous SCD, exploring these measures in patients with other genotypes is also vital and may help shed further light on the underlying physiology. Finally, further work is also required to establish the applicability of this framework to other end-organs which are also at risk of damage in SCD.

If fruitful, this line of enquiry has the potential to improve precision medicine in SCD, which is a crucial next step in efforts to screen and intervene. Whilst there are evidence-based strategies for stroke prevention in children (10), treatment is often burdensome (305), the specificity of screening is poor (10), and many patients continue to suffer progressive vasculopathy and/or recurrent insults (38). There are few evidence-based strategies for cognitive dysfunction, and none that tackle microstructural tissue injury. Improved strategies are therefore urgently required.

According to the proposed framework, measures of regional oxygen delivery, CVR, and OEF are likely the most proximate targets for prediction of neurological risk. With further refinement, development of a “vascular instability risk profile” based on these measures may enable selection of patients with sufficiently high-risk for invasive, burdensome, and costly treatment options such as transfusion, bone marrow transplant, or eventually gene therapy. Such a profile may also enable on-going monitoring of risk so that transfusion is not necessarily lifelong. Another implication is the identification of regional oxygen delivery as a potential treatment target for therapies, with several potential avenues for intervention (e.g., anemia, oxygen desaturation, endothelial dysfunction). Crucially, therapies need to balance any increases in oxygen-delivery with any potential reductions in oxygen-unloading (293). With further refinement, this framework may therefore hold promise not only for guiding research, but also for prediction of risk and implementation of tailored preventative strategies before stroke, SCI and/or microstructural injury occurs.

## Author Contributions

HS and FK: design and conception. HS, FK, and JK: literature review. HS: drafting the article. FK, JK, PH, DS, and CC: critical revision of the article. HS, FK, JK, PH, DS, and CC: final approval of the version to be published.

### Conflict of Interest Statement

The authors declare that the research was conducted in the absence of any commercial or financial relationships that could be construed as a potential conflict of interest.
